# Immune cells subpopulations in cerebrospinal fluid and peripheral blood of patients with Aneurysmal Subarachnoid Hemorrhage

**DOI:** 10.1186/s40064-015-0970-2

**Published:** 2015-04-23

**Authors:** Leandro Moraes, Sofía Grille, Paula Morelli, Rafael Mila, Natalia Trias, Andreína Brugnini, Natalia LLuberas, Alberto Biestro, Daniela Lens

**Affiliations:** Cátedra de Medicina Intensiva. Hospital de Clínicas. Facultad de Medicina, Universidad de la República, Montevideo, Uruguay; Cátedra de Hematología. Hospital de Clínicas. Facultad de Medicina, Universidad de la República, Avda. Italia s.n, CP 11300 Montevideo, Uruguay; Departamento Básico de Medicina, Hospital de Clínicas, Facultad de Medicina, Universidad de la República, Montevideo, Uruguay; Departamento de Cardiología. Hospital de Clínicas. Facultad de Medicina, Universidad de la República, Montevideo, Uruguay

**Keywords:** Neuroinflammation, Cerebrospinal fluid, Aneurysmal subarachnoid hemorrhage, Systemic inflammation, Flow cytometry

## Abstract

**Background:**

There is growing evidence supporting the role of inflammation in aneurysmal subarachnoid hemorrhage (aSAH) pathophysiology and it is of great interest to elucidate which immune mechanisms are involved.

**Methods:**

12 aSAH patients and 28 healthy controls were enrolled prospectively. We assessed leukocytes subpopulations and their activation status by flow cytometry in cerebrospinal fluid (CSF) and peripheral blood (PB) of SAH patients at the same time and in PB of controls.

**Results:**

Monocytes and neutrophils were activated in CSF of aSAH patients. The percentage of CD14^++^CD16^+^ monocytes were higher in CSF than in PB of aSAH patients, and were also increased in PB of aSAH patients compared with controls. An enhanced expression of CD69 was shown in CSF neutrophils compared with PB in aSAH patients. PB of aSAH patients showed lower percentage of total lymphocytes compared with controls PB. Additionally, lymphocytes were activated in CSF and PB of aSAH patients. CD4^+^ and CD8^+^ T cells had a decreased expression on CD3 and higher levels of CD69 in CSF compared with PB in aSAH patients. Moreover, PB CD4^+^ and CD8^+^ T cells of aSAH patients were activated compared with controls. Additionally, CD28 expression was decreased on CSF T lymphocytes.

**Conclusions:**

Our data suggest an important recruitment of leukocytes to the site of injury in aSAH as well as an increased activation at this level. Overall, these results indicate that aSAH probably stimulates both the innate and adaptive immune responses.

## Background

Aneurismal subarachnoid hemorrhage (aSAH) is an acute cerebrovascular event which can have catastrophic impact not only on the central nervous system but also on several other organs (Hinson and Sheth 2012; Mashaly and Provencio 2008; Schuiling et al. 2005; Stevens and Nyquist 2007). Despite great advances in neuroscience that had occurred in the last decades, morbidity and mortality remain very high. There is growing evidence for the role of inflammatory response in the pathophysiology of main aSAH complications (intracranial hypertension, rebleeding and vasospasm) (Miller et al. 2014); (McMahon et al. 2013); (Provencio and Vora 2005); (Provencio 2013).

It has been postulated that the extravasated blood in the subarachnoid space following aneurysm rupture is responsible for a cascade of immune reactions involving the release of various vasoactive and pro-inflammatory factors which contributes to early and delayed brain injury (Caner et al. 2012; Edvinsson and Povlsen 2011; Fujii et al. 2013; Lindgren et al. 2014; Mehta et al. 2013; Provencio 2013; Wan et al. 2014). Many results strongly suggest that aSAH elicits an immunological process that includes both cellular and humoral immunity (Chen et al. 2014; Plog et al. 2014; J J Provencio et al. 2010; Provencio 2013; Xie et al. 2013). Perivascular leukocyte accumulation has been demonstrated in both experimental and clinical models of SAH (Schneider et al. 2012; Sercombe et al. 2002). Recent studies showed a pro-inflammatory milieu in serum and cerebrospinal fluid (CSF) after the hemorrhagic event (Schneider et al. 2012). Pro-inflammatory cytokines such as interleukin-6 (IL-6), IL-1, TNF-α and complement have been detected in CSF of aSAH patients indicating that they might be involved in the pathogenesis of vasospasm (Dhar and Diringer 2008; Dumont et al. 2003; Fassbender et al. 2001; Greenhalgh et al. 2012; Hendryk et al. 2004; Kwon and Jeon 2001; Sarrafzadeh et al. 2010; Sercombe et al. 2002; Xie et al. 2013; You et al. 2013; Zhou et al. 2007). Little is known about the role of adaptive immune system in aSAH. Necropsies and studies in animals of the blood vessels and walls of ruptured aneurysms have found B and T lymphocyte infiltration in the wall of aneurysm tissue (Chyatte et al. 1999; Hughes and Schianchi 1978). However, analysis of T and B cell subsets in CSF and peripheral blood (PB) in aSAH patients is not yet available.

In this report, we studied and compared in paired samples of CSF and PB, granulocytes, monocytes and lymphocytes subsets and their activation status in twelve aSAH patients and controls. These findings may contribute to the knowledge of the immunopathological mechanisms underlying early and late brain injury in aSAH and may promote the development of novels treatment strategies (Chen et al. 2014; Cheng et al. 2014; Echigo et al. 2012; Muroi et al. 2014; Satoh et al. 2014; Uekawa et al. 2014; Zhang et al. 2014).

## Results

### Patient characteristics

Clinical and demographic characteristics of the twelve enrolled patients and controls are shown in Table 1. 75% (n = 9) of patients presented with Hunt and Hess (HH) grade of 4 or above and the majority had Fisher grade 3 SAH (n = 7). CSF and PB samples were obtained within 6 days from bleeding (day 0). Five patients (42%) developed vasospasm and seven died (58%).

**Table 1:** **Clinical and demographic characteristics**

	**aSAH patients (n = 12)**	**Controls (n = 28)**	**p value**
**Age (median and range)**	48.5 (34–67)	45 (25–76)	0.62 (NS)
**Gender. Male: female**	0.33:1	0.1:1	0.46 (NS)
**Hunt & Hess score**			
**1**			
**2**	1		
**3**	2		
**4**	1		
**5**	8		
**Fisher scale**			
**1**	1		
**2**		-	-
**3**	7		
**4**	4		
**Coils(n)/ Clips(n)**	6/6		
**Vasospasm n (%)**	5 (42)		
Mortality n* (%)	7/12 (58)		

*In the intensive care unit.

### CSF white cell count

We have measured the absolute number of white cells in CSF of SAH patients prior flow cytometry analysis. All CSF samples contained an increase and sufficient number of leukocytes for flow cytometry analysis. We found an absolute white cell count mean of 745,4×10^6^/l (range 100–3100×10^6^/l).

### Monocytes and neutrophils were activated in CSF of aSAH patients

**Figure 1:**
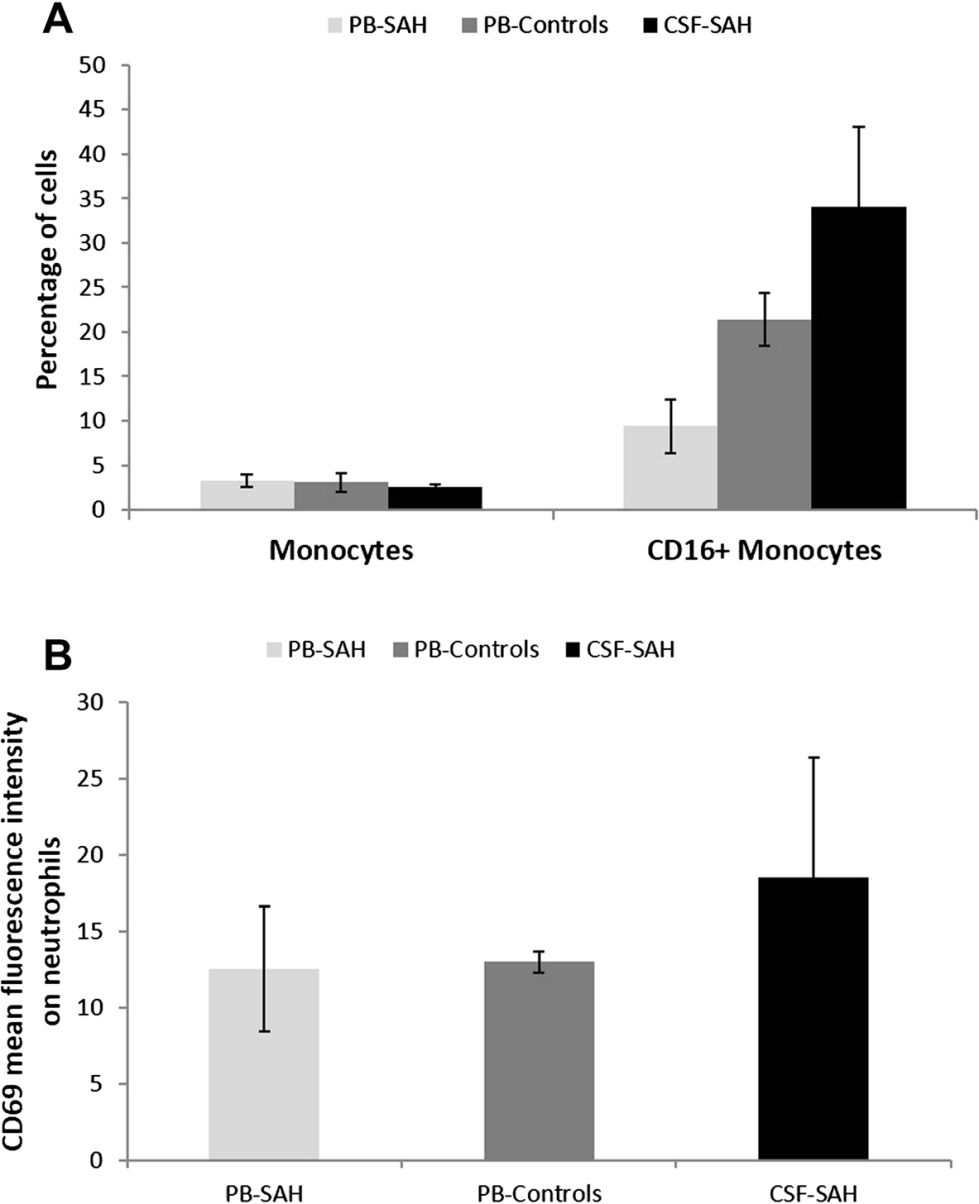
Monocytes and neutrophils. **A)** Percentages of total monocytes and CD14^++^CD16+ monocytes in CSF and PB of aSAH patients and in PB of controls. Data shown are median ± standard error. **B)** CD69 expression on neutrophils in CSF and PB of aSAH patients and in PB of controls. Data shown are median of CD69 mean fluorescence intensity (MFI) on neutrophils ± standard error.

As shown in Figure 1A the percentage of CD14^++^CD16^+^ monocytes in CSF (median 34%) were higher than those in PB of aSAH patients (median 9.4%). Also, CD14^++^CD16^+^ monocytes were lower in PB of aSAH patients compared with controls (median 21.4%; p = 0.010). No differences were observed in total monocytes percentage.

No differences were observed in neutrophils percentage between groups. However, the proportion of neutrophils expressing the early activation marker CD69 was higher in CSF than in PB in aSAH patients (Figure 1B).

### Lymphocytes subsets in CSF and PB of aSAH patients and controls

**Figure 2:**
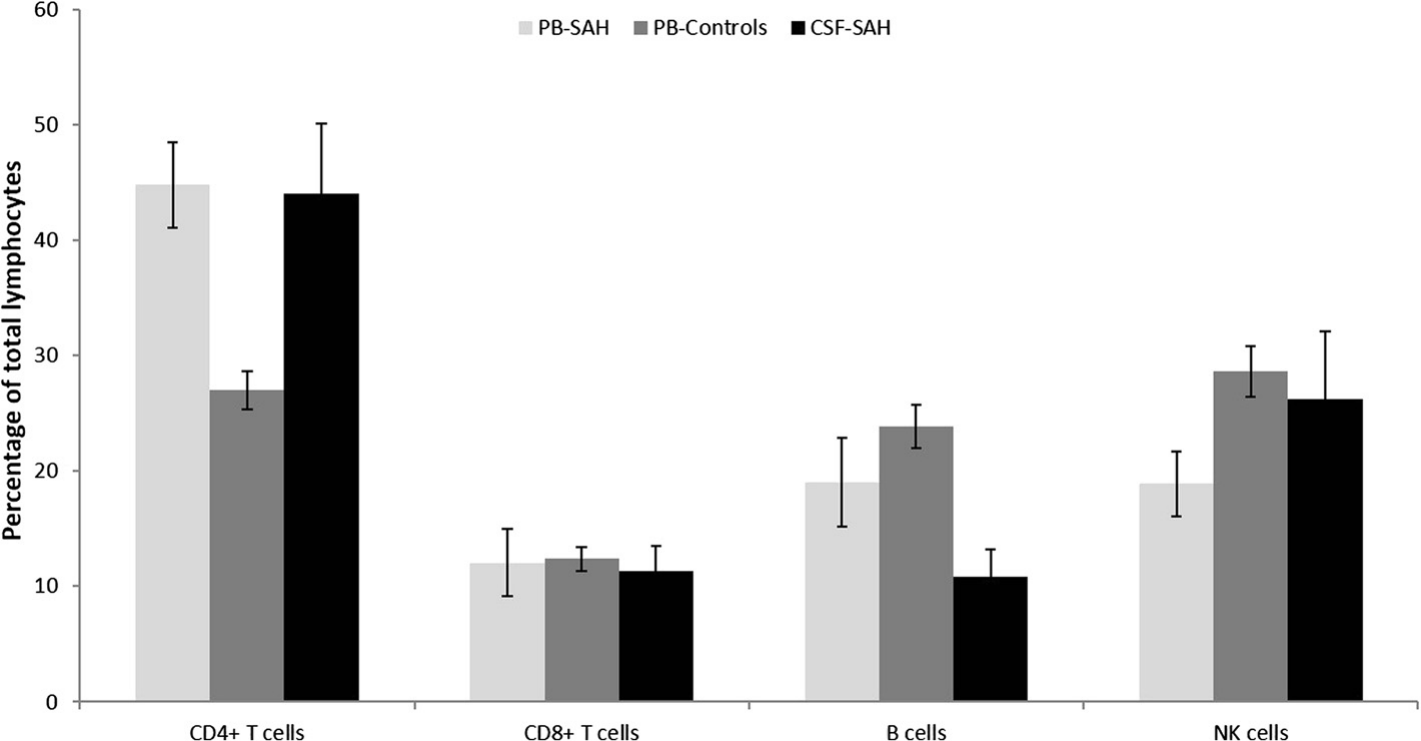
Lymphocytes subsets. Percentages of CD4^+^ T cells, CD8^+^ T cells, NK cells and B cells lymphocytes of total lymphocytes in CSF and PB of aSAH patients and in PB of controls. Data shown are median ± standard error.

The percentage of CD4^+^ T cell, CD8^+^ T cells, B and NK cells of total lymphocytes were similar between CSF and PB of aSAH patients. PB of aSAH patients showed lower percentage of total lymphocytes compared with controls. In addition, differences in the distribution of lymphocyte subpopulations were observed. As shown in Figure 2, PB of aSAH patients showed a higher percentage of CD4^+^ T cells and a lower percentage of NK cells compared with controls. Additionally, the percentage of B cells was slightly lower in CSF compared with PB of aSAH patients.

### Lymphocytes were activated in CSF and PB of aSAH patients

**Figure 3:**
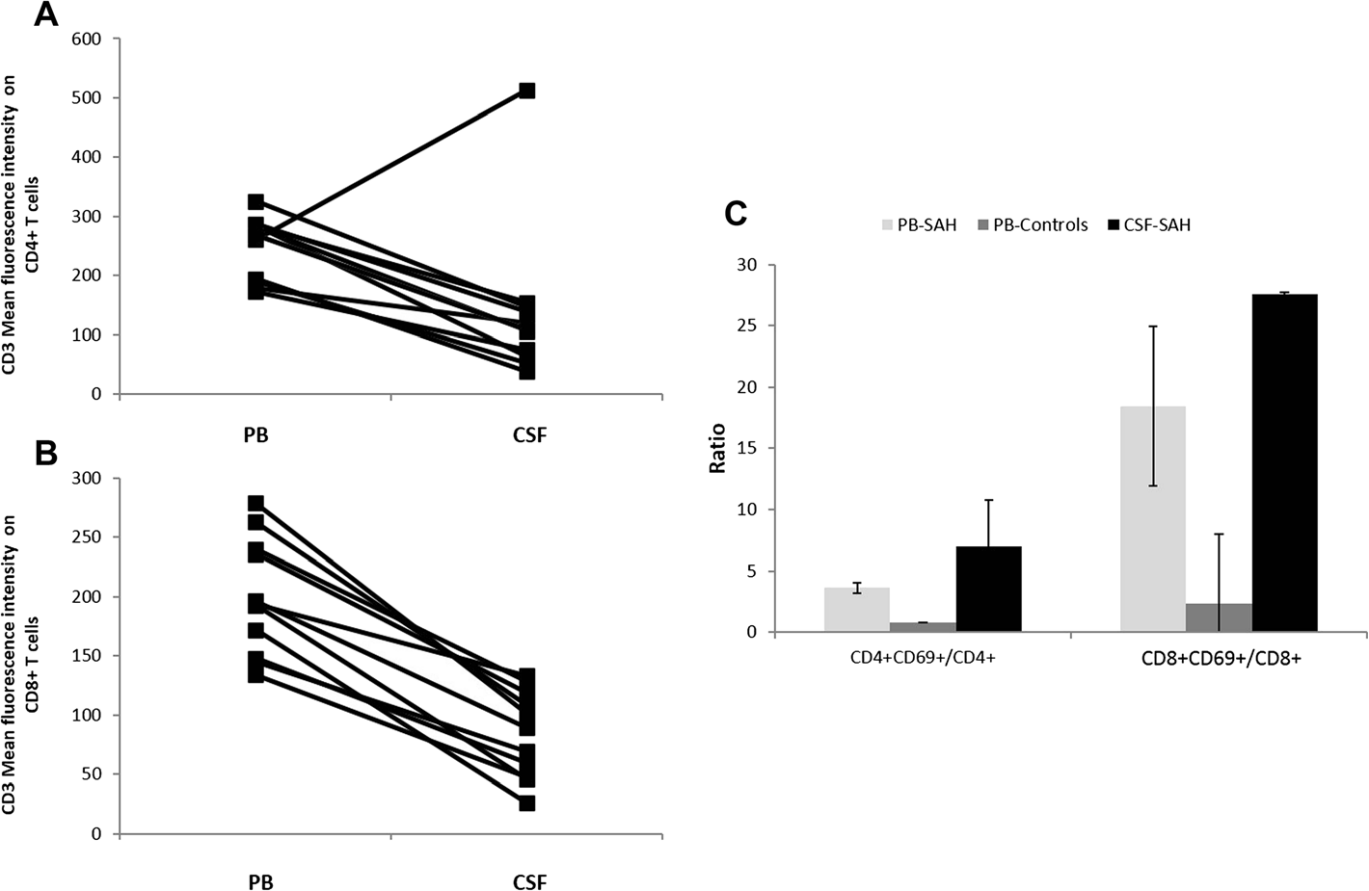
Activation pattern in CD4^+^ and CD8^+^ T cells. **A)** CD3 mean fluorescence intensity (MFI) on CD4^+^ T cells in PB and CSF of aSAH patients. Lines indicate samples from the same patient on the same time. **B)** CD3 MFI on CD8^+^ T cells in PB and CSF of aSAH patients. Lines indicate samples from the same patient on the same time. **C)** Ratio of CD4^+^ CD69^+^ T cells/CD4^+^ T cells and CD8^+^ CD69^+^ T cells/CD8^+^ T cells. Data shown are median ± standard error.

CD4^+^ and CD8^+^ T cells showed an activated profile (Figure 3). As shown in Figure 3A and B, CD4^+^ and CD8^+^ T cells had a decreased expression on CD3 in CSF compared with PB in aSAH patients. We observed almost a 1,8 fold decrease in CD3 expression on CD4^+^ T cells and a twofold decrease on CD8^+^ T cells in CSF compared with PB sampling at the same time. Additionally, the ratio of activated CD4^+^/Total CD4^+^ T cells (CD4^+^CD69^+^/CD4^+^) and activated CD8^+^/ Total CD8^+^ T cells (CD8^+^CD69^+^/CD8^+^) were increased in CSF compared with PB in aSAH patients (Figure 3C). Moreover, PB CD4^+^ and CD8^+^ T cells of aSAH patients were activated compared with controls. As shown in Figure 3C, CD4^+^CD69^+^/CD4^+^ and CD8^+^CD69^+^/CD8 were increased in aSAH patients compared with controls.

### CD28 expression was decreased on CSF T lymphocytes

**Figure 4:**
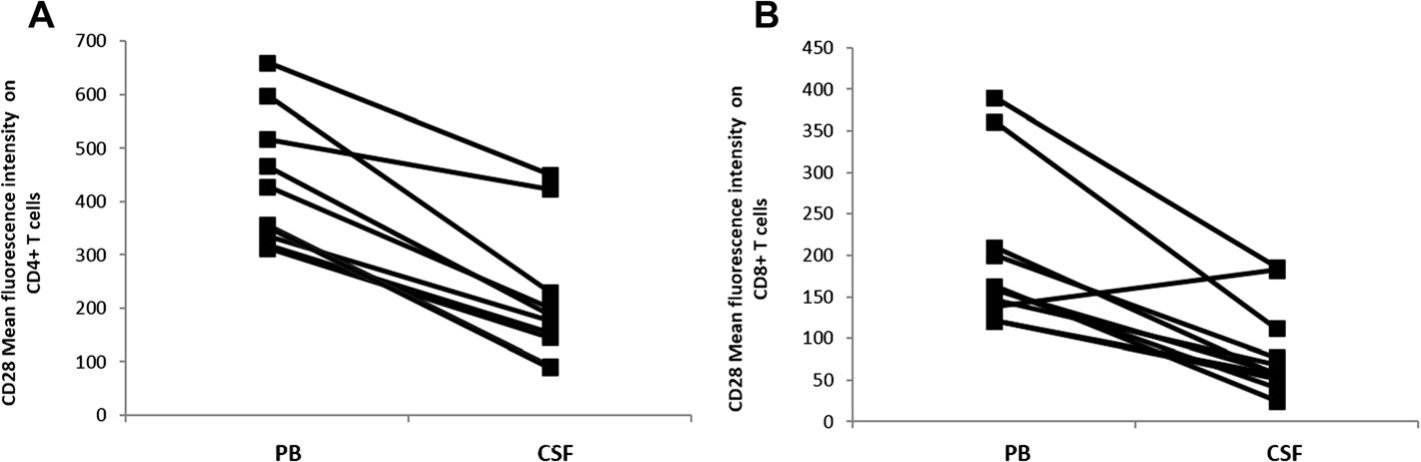
CD28 expression on CD4^+^ and CD8^+^ T cells. **A)** CD28 mean fluorescence intensity (MFI) on CD4^+^T cells of PB and CSF of aSAH patients. Lines indicate samples from the same patient on the same time. **B)** CD28 MFI on CD8^+^T cells on PB and CSF of aSAH patients. Lines indicate samples from the same patient on the same time.

We also studied the expression of T-cell co-stimulatory molecule CD28 on CD4^+^ and CD8^+^ T cells. As shown in Figure 4, CSF CD4^+^ and CD8^+^ T cells express lower levels of CD28 than PB CD4^+^ and CD8^+^ T cells in aSAH patients. We observed a twofold decrease in CD28 expression on CD4^+^ T cells and a 2.7 fold decrease on CD8^+^ T cells in CSF compared with PB sampling at the same time. No differences were observed in CD28 expression of CD8+ T cells between PB of aSAH patients and controls.

In five patients we took 2 CSF samples in different moments (1 to 3 and 4 to 6 days from bleeding). No differences among any subtype of immune cell subpopulations (including activation status) were evidenced (data not shown). Five patients had an EVD and a LD inserted during the first days after bleeding. CSF samples from both places were taken in two different moments (48 hours interval) simultaneously. No differences among any subtype of immune cell subpopulations (including activation status) were evidenced (data not shown).

## Discussion

Aneurysm rupture provokes a substantial local and systemic inflammatory response and there is convincing evidence that inflammation plays a critical role in early and delayed brain injury contributing to the poor outcome caused by SAH (Chou et al. 2012; Rodríguez-Rodríguez et al. 2014; Sercombe et al. 2002).

Blood cells, cytokines and complement in the subarachnoid space following aneurysm rupture seem to play a major role triggering inflammatory reactions. Cytokines can trigger strong pro and anti-inflammatory effects. Inflammatory cytokines in CSF, such as IL-6, IL-8, IL-1 and TNF-α were shown to be elevated in aSAH patients, with a greater increase in patients with unfavourable outcome (Chou et al. 2012; Fassbender et al. 2000; Gruber et al. 2000; Hirashima et al. 1997; Nakahara et al. 2009; Provencio 2013; Sarrafzadeh et al. 2010).

This highlights the involvement of innate immunity in SAH. In addition, several reports have linked CSF cytokines increase with the generation of delayed brain injury associated with vasospasm, one of the most common and feared complications of aSAH (Bowman et al. 2006; Hendryk et al. 2004; Ni et al. 2011; Provencio 2013; Wang et al. 2007; You et al. 2013)

In this report we show an activated profile in innate immune cells (monocytes and neutrophils). We found a marked increase in the percentage of CD14^++^CD16^+^ monocytes in CSF compared with PB and a decreased in PB of aSAH patients compared with controls. Human blood monocytes are heterogeneous and conventionally subdivided into three subsets based on CD16 and CD14 expression (CD14^++^CD16^−^, CD14^+^CD16^++^, CD14^++^CD16^+^ monocytes) (Wong et al. 2012). It is known that CD16^+^ expression in monocytes/ macrophages indicates activation of these cells and it has been suggested that they have a proinflammatory function based on higher expression of proinflammatory cytokines and higher potency in antigen presentation (Ziegler-Heitbrock 2007). Belge et al. found that after *in vitro* stimulation with LPS, CD16^+^ monocytes produced high levels of TNF-α (pro-inflammatory cytokine) and low levels of IL-10 (anti-inflammatory cytokines) (Belge et al. 2002). Many reports showed that TNF-α was increased in CSF of aSAH patients, particularly in patients with unfavorable outcomes (Chou et al. 2012; Fassbender et al. 2000; Kwon and Jeon 2001; Mathiesen et al. 1997). Although a variety of cell types are known to express TNF-α, CD16^+^ monocytes could be an important source of TNF-α in CSF of aSAH patients. Additionally, it has been published that CD16^+^ monocytes show a higher level of HLA-DR (Passlick et al. 1989), and this would predict a higher antigen presenting cell (APC) activity in these cells. *In vitro* studies found that CD16^+^ monocytes was three times more potent APC when compared with CD16^−^ monocytes (Grage-Griebenow et al. 2001; Zawada et al. 2012). Our results evidence that CD14^++^CD16^+^ monocytes infiltrate the CSF of aSAH patients suggesting that they may play an important role in the pathogenesis of this disease.

To study the activation status of neutrophils, we measured the CD69 surface activation marker (very early activation antigen) expression. We found an increased (CD69 expression) in CSF neutrophils compared with PB of aSAH patients, suggesting an increased activation status in CSF neutrophils. Activated neutrophils could not only participate in subarachnoid blood clearance, but also could be an important source of cytokines in CSF of aSAH patients (Cassatella 2013).

The increased proportion of CD69^+^ neutrophils and CD14^++^CD16^+^ monocytes probably indicates an ongoing systemic inflammation and a stimulation of innate immunity in aSAH patients.

On the other hand, we also studied and found interesting data about adaptive immune cells. T lymphocytes are central in developing a sustained inflammatory response, and they are source of cytokines that may play a role in early and delayed brain injury after aSAH. Although no difference were found in percentage of CD4^+^ and CD8^+^ T cells between CSF and PB of aSAH patients and controls, CSF CD4^+^ and CD8^+^ T cells showed an increased activation status determined by decreased expression of CD3, CD28 and an increased expression on CD69. Additionally, PB CD4^+^ and CD8^+^ T cells were more activated compared with controls, assessed by CD69 expression. CD3 downregulation from the cell surface is normally associated with *in vitro* and *in vivo* T-cell activation (Comar et al., 2005; Sullivan and Coscoy, 2008). We also study the expression of T-cell co-stimulatory molecule CD28 on CD4^+^ and CD8^+^ T cells. We found that CSF CD4^+^ and CD8^+^ T cells express lower levels of CD28 than PB CD4^+^ and CD8^+^ T cells in aSAH patients. CD28 is the only B7 receptor constitutively expressed on naive T cells and provides co-stimulatory signals, which are required for T cell activation. Stimulation through CD28 and T Cell Receptor (TCR) can provide a potent co-stimulatory signal to T cells for the production of various interleukins. Human T-cells that have undergone repeated cycles of antigen-driven proliferation develop a series of phenotypic and functional changes, including the disappearance of cell-surface CD28. Hence, finding a lower expression of CD28 or CD4 + CD28null cells suggest the presence of an activation status and a chronic adaptive immune response (Studer et al. 2008; Vallejo et al. 2004; Vallejo 2005; van Leeuwen et al. 2004). Additionally, it has been reported that TNF-α downregulated the expression of CD28 on T cells (Bryl et al. 2001). This could explain the finding of the increased CD14^++^CD16^+^ monocytes number and the increased level of TNF-α previously reported in these patients.

Adaptive immunity has been little studied in the context of aSAH. Mathiesen et al. in 1993 reported that IL-2 receptor and soluble CD8 levels were increased in CSF of SAH patients suggesting the role of adaptive immune response in the SAH pathogenesis (Mathiesen et al. 1993). Adaptive immunity has been study in ischemic stroke, in ischemia-reperfusion brain injury, and other neurologic disease (Brait et al. 2012; Cepok et al. 2001). Following an ischemic stroke, T cells become activated, infiltrate the brain and release cytokines and reactive oxygen species that probably contribute to brain injury (reviewed in (Brait et al. 2012)). Additionally, there is evidence that T cells accumulate in the post-ischemic brain within few hours after reperfusion (reviewed in (Brait et al. 2012)). As far as we know, this is the first report that evidences an early activation profile in CSF CD4^+^ and CD8^+^ T cells in aSAH patients, suggesting the participation of adaptive immune response in the immunopathogenesis of this disease.

We also found that the percentage of NK and B cells in PB of aSAH patients were lower compared with controls, but without an increase in CSF compared with PB of aSAH patients. In other disease models, for example in ischemic stroke, it has been observed that the levels of lymphocytes and other immune effectors in circulation are reduced, possibly as an endogenous protective mechanism (Liesz et al. 2009; Martin et al. 2008). Also, there are a reduced number of T cells and other immune cells in spleen, thymus and lymph nodes (reviewed in (Brait et al. 2012)). Some authors postulate that this determines a “stroke-induced immunodeficiency syndrome” that may contribute to the increased incidence of infections observed in these patients. Urra et al. reported an increased apoptosis rate and a reduction in PB levels of CD4^+^ and CD8^+^ T cells, T regulatory cells and B cells following ischemic stroke (Urra et al. 2009). There is increasing evidence that an impairment of cellular immune function after acute central nervous system injury, such as stroke and traumatic brain injury represents a risk factor for infections (Chamorro et al. 2007; Klehmet et al. 2009; Woiciechowsky et al. 1998). Additionally, Sarrafzadeh et al. reported a SAH-induced immunodepression and found T-lymphopenia and a decreased monocyte human leukocyte antigen-DR expression on PB. Immunodepression was associated with a high incidence of pneumonia (Sarrafzadeh et al. 2011). In that way, we may speculate that the slightly reduction in the percentage of PB NK and B cells could be due to an increase in apoptosis or a differentiation to other subpopulation probably contributing to the immunosuppression observed in this patients.

### Limitation of the study

The main limitation of our pilot study is the small number of enrolled patients. Thus, we decided not to attempt a detailed statistical analysis. Additionally, considering the small sample of patients and the fact that most of them have a severe aSAH (Table 1) with high mortality our results should be interpreted with caution and could not be extrapolated to patients suffering from less severe disease. Further investigation is warranted.

Additionally, in our hospital EVDs and/or LDs are inserted in the acute period (within the first week from bleeding) mainly in patients complicated with hydrocephalus, Fisher III or IV (increased risk of vasospasm) and/or GCS < 9 (poor clinical grade with greater risk of vasospasm and intracranial hypertension). The enrollment of a significant number of mild cases (HH 1–3 and/or Fisher 2) would only be achieved if new and convincing data arises in the near future from ongoing trials (Early drain trial is not enrolling Fisher 1 patients).

Although we did not find any differences among immune cells subpopulations and their activation status in two different moments within the first week since aneurysm rupture (day 0), our approach only covers a very short period of time and changes are still possible considering that the inflammatory response is essentially a dynamic process.

Finally, the risk of infection represents a clear obstacle to extend the sampling period at our institution (the infection rate increases substantially when an EVD stays longer than 5 days and a LD stays longer than 7 days). The introduction of new coated catheters could help us to overcome this critical barrier.

## Conclusion

The results presented here suggest that effectors of both innate and adaptive immune response may play a role in the pathogenesis of severe aSAH. We found not only a recruitment in CSF and activation of monocytes and neutrophils (innate immune response effectors), but also an activation of CD4^+^ and CD8^+^ T cells (adaptive immune response effectors). More research and a greater number of patients evaluated are needed to gain greater understanding in the immunopathogenesis of aSAH and to possibly identify immune biomarkers of severity and therapeutic targets.

### Patients and methods

#### Patients and control

aSAH patients and control subjects were recruited prospectively. Twelve patients (9 women and 3 men) with a median age of 48.5 years (range 34–67 years) with aSAH were included in the study. Twenty eight healthy control subjects (asymptomatic, without any major vascular risk factor or acute or chronic inflammatory/infection disease) were included with a median age of 45 (range 25–76 years). There were no significant differences in age between aSAH and control group (Table 1).

Considering recent published data (Kowarik et al. 2014) we decided not to perform CSF flow cytometry analysis from healthy controls. Percentages of immune cells main subtypes (CD4+ T cells, CD8+ T cells, CD4/CD8 ratio, monocytes, B cells, plasmablasts and NK cells) in normal CSF from symptomatic controls (headache and paresthesia of unspecific origin) as a reference are reported.

Depending on hospital availability, the aneurysm diagnostic technique were digital subtraction angiography (DSA) or computed tomography angiography (CTA). Patients with traumatic SAH, pregnancy, intracranial malignancies, or infectious meningitis were excluded.

All SAH patients underwent transcranial doppler (TCD) monitoring for vasospasm screening from day 1 to 14. Vasospasm in the middle cerebral artery (MCA) and anterior cerebral artery (ACA) was defined by a mean cerebral blood flow velocity (mCBFV) exceeding 120 cm/s and three times the mean flow velocity (FV) of the ipsilateral extracranial internal carotid artery (eICA) (Lindegaard Ratio > 3). Basilar artery (BA) vasospasm was defined whenever the mCBFV was higher than 85 cm/s and two times the mean FV of the extracranial vertebral artery (eVA)(Soustiel Ratio > 2).

#### Sampling

CSF and PB samples were collected within 6 days from bleeding (median 3 days, range 1–6 days). CSF samples were acquired from patients who had an external ventricular drain (EVD) and/or a lumbar drain (LD) placed for clinical indications. At the same time, 1 ml of CSF and 2 ml of PB were collected from aSAH patients. Automated CSF leukocyte counts were obtained on ABX micros 60 (Horiba, Montpellier, France). Samples were examined by 4-colour flow cytometry within 30 min from sampling. Additionally, 2 ml of PB were collected from controls.

#### Leukocyte subsets in cerebrospinal fluid and peripheral blood

Analyses of leukocyte subsets were conducted by flow cytometry on CSF and PB taken from patients and controls. Cells were washed twice with phosphate-buffered saline and immunostained for 15 min at room temperature with the following panel of antibodies: PE-Cy5.5-conjugated anti-CD3, APC-conjugated anti-CD3, APC-conjugated anti-CD4, APC-conjugated anti-CD14, FITC-conjugated anti-CD4, FITC-conjugated anti-CD8, APC- conjugated anti-CD8, PE-conjugated anti-CD69, PE-conjugated anti-CD28, FITC-conjugated anti-CD56, PE-conjugated anti-CD16, PERCP-conjugated anti-CD19, FITC-conjugated anti-CD16, FITC-conjugated CD11b (all reagents from BD Pharmingen, San Diego, USA). Optimal antibody concentrations were previously defined by titration. After incubation, red blood cells were lysed with FACS lysing solution (Becton-Dickinson, San Diego, CA). The cells were washed and suspended in phosphate-buffered saline. Flow cytometry data was collected on a FACS Calibur Flow cytometer equipped with two lasers (Becton–Dickinson, Oxford, UK). For data acquisition and analysis CellQuest software (Becton–Dickinson) and Infinicyt™ (Cytognos, Spain) were used. Gating strategy: neutrophils were gated by side scatter and CD11b. Monocytes were gated by side scatter and CD14, subsequently CD16 and CD69 were evaluated. T cells were gated by side scatter and CD3 and subsequently by CD8 or CD4. Then, CD69 and CD28 expression were measured. B cells were gated by side scatter and CD19 expression without CD3 expression. NK were gated by side scatter and CD56 or CD16 expression and different subsets were measured.

#### Statistical analysis

Statistical analysis was performed using SPSS (Chicago, IL, USA). Descriptive statistics were used and measures of central tendency and dispersion were calculated.

#### Ethical consideration

Informed consent was obtained from the patients or relatives and controls enrolled in this study. The protocol was approved by the Institutional Review Board of de Hospital de Clínicas, Montevideo, Uruguay, in accordance with the Declaration of Helsinki.

## Abbreviations

ACA: Anterior cerebral artery
APC: Antigen presenting cell
aSAH: Aneurysmal subarachnoid hemorrhage
BA: Basilar artery
CSF: Cerebrospinal fluid
CTA: Computed tomography angiography
DSA: Digital subtraction angiography
elCA: Extracranial internal carotid artery
eVA: Extracranial vertebral artery
EVD: External ventricular drain
FV: Flow velocity
HH: Hunt and hess
LD: Lumbar drain
MCA: Middle cerebral artery
mCBFV: Mean cerebral blood flow velocity
MFI: Mean fluorescence intensity
PB: Peripheral blood
TCD: Transcranial Doppler
TCR: T cell receptor
